# Thyroid dysfunction in pregnancy - a retrospective observational analysis of a Maltese cohort

**DOI:** 10.1186/s12884-022-05266-x

**Published:** 2022-12-15

**Authors:** Katia Vella, Sandro Vella, C. Savona-Ventura, J. Vassallo

**Affiliations:** 1 Department of Obstetrics and Gynaecology, Mater Dei Hospital, Msida, Malta; 2 Department of Obstetrics and Gynaecology, Faculty of Medicine and Surgery, University of Malta Medical School, Msida, Malta; 3 Department of Medicine, Faculty of Medicine and Surgery, University of Malta Medical School, Msida, Malta; 4 Department of Medicine, Division of Endocrinology, Mater Dei Hospital, Msida, Malta

**Keywords:** Thyroid, Pregnancy, Prevalence, Outcomes

## Abstract

**Background:**

Thyroid dysfunction is known to adversely affect pregnancy. This study evaluates the prevalence of thyroid disorders and explores their association with pregnancy complications/comorbidities and modes of delivery in the Maltese pregnant population over a ten year period.

**Design:**

A population based observational study.

**Method:**

We analysed data from the National Obstetrics Information Service of the Department of Health Informations and Research (NOIS) for all births delivered in Malta between 2006 and 2016. Cases identified and recorded by NOIS to have had some form of thyroid dysfunction during pregnancy were confirmed by cross-referencing  with laboratory results found in patients’ medical records and/or iSOFT® database system. Using the Statistical Package for the Social sciences (SPSS®)  demographic data, past obstetric and medical history and obstetric outcomes were analysed for pregnancies with thyroid dysfunction and compared to data pertaining to pregnancies in euthyroid patients, that is those with no recorded thyroid dysfunction on NOIS. Chi square/Fisher's exact test were used to compare categorical variables while ANOVA/Mann-Whitney U test was used to compare continuous variables. Statistical significance was defined by a two-sided *p* value <0.05.

**Results:**

Data was available for 46,283 women (mean [SD] age = 29.2 [5.4] years). 587 pregnancies (1.3%) suffered from thyroid dysfunction. Of these, 67.3% were hypothyroid, 3.2% had hyperthyroidism, 28.3% had isolated hypothyroxinaemia (IHT) while 1.2% had a history of thyroid carcinoma. Patients with IHT and hypothyroidism were older than euthyroid patients (*p* < 0.001).  IHT and hypothyroid patients had a statistically significant higher body mass index (BMI) than euthyroid women (*p*=0.001 for hypothyroid women, *p* = 0.035 for IHT). Hypothyroid  and IHT women were more likely to have had a previous lower segment caesarean section (*p*=0.043,  and 0.006 respectively). Type 1 diabetes and gestational diabetes  *p* = 0.012) were more common associated comorbidities in hypothyroid pregnancies. Offspring of patients with IHT had a higher birth weight than those born to euthyroid patients (*p*=0.009). Patients with hyperthyroidism were found to have a significantly increased risk of early preterm delivery before 34 weeks of gestation and were also more likely to have suspected intrauterine growth restriction and low mean birth weight. We report no significant differences in past history of obstetric loss, antenatal complications, mode of delivery, gestational age at delivery and postpartum haemorrhage rates across thyroid categories.

**Conclusions:**

Available evidence suggests that thyroid dysfunction is more likely in the setting of older age, and higher body mass index. Moreover, it impacts on neonatal birth weight, rates of early preterm delivery and intrauterine growth restriction.

**Supplementary Information:**

The online version contains supplementary material available at 10.1186/s12884-022-05266-x.

## Background

Maintaining euthyroidism in the pregnant mother is important for healthy foetal growth and development. Even minor changes in maternal thyroid function can cause detrimental effects for the foetus in addition to potential adverse effects on the pregnancy itself [1].

Primary hypothyroidism, generally defined as the presence of elevated Thyroid Stimulating Hormone (TSH) concentrations, is classified as overt hypothyroidism if serum Thyroxine (Ft4) levels are low and subclinical hypothyroidism if serum Ft 4 levels are within normal limits. While hypothyroidism affects 2–3% of all pregnant women, overt hypothyroidism affects between 0.2 and 1% of all pregnancies [2]. Untreated overt hypothyroidism in pregnancy has consistently been shown to be associated with adverse effects on the maternal-foetal unit namely increased risk of prematurity, low birth weight, intrauterine growth restriction, increased risk of gestational hypertension and increased risk of foetal loss as well as increased risk of neurocognitive deficits in the developing foetus [1].

The prevalence of subclinical hypothyroidism (SCH) is known to vary depending on ethnicity, iodine intake and definition. Historically it was reported to range between 2 and 3% [1]. The redefinition of the upper limit of TSH levels during pregnancy to 2.5mIU/L in the first trimester and 3.0 to 3.5 mIU/ in the second and third trimesters of pregnancy resulted in an increase in the prevalence rates of women with SCH in pregnancy with studies reporting prevalence rates of 6.8% [3] and 15% [4].

Several studies have suggested that even SCH during pregnancy can affect foetal neuropsychological development and be associated with adverse pregnancy outcomes namely an increased risk of pregnancy loss, placental abruption, perinatal mortality, admission to the neonatal intensive care unit, low Apgar scores, pre-term delivery and low birth weight [1, 5, 6]. The Generation R study, however, failed to confirm an association between SCH and pre term deliveries when TPOAb + ve women were excluded [7]. Other studies and meta- analysis have suggested an increased risk of Gestational Diabetes (GDM) in patients with hypothyroidism [8, 9] while other studies have found a significant association with gestational hypertension and pre-eclampsia [10, 11].

The presence of serum T4 (TT4 or FT4) concentrations in the lower 2.5-5% of the reference range for a given population with normal TSH concentrations is defined as Isolated Hypothyroxinaemia [12]. Several prospective, nonrandomised studies have reported adverse outcomes namely neurocognitive impairment, worsened motor function and smaller head circumference in children born to mothers with isolated hypothyroxinaemia [13–15] Other studies have reported an association between isolated hypothyroxinaemia and adverse pregnancy outcomes including pre-term delivery [7].

Hyperthyroidism results when the serum concentrations of T4 and/or free triiodothyronine (T3) are high. During pregnancy, the commonest cause of hyperthyroidism is Graves’ disease. Hyperthyroidism affects 0.2% of pregnancies and has been associated with increased risk of miscarriages, gestational hypertension, prematurity, low birth weight, intrauterine growth restriction, stillbirth, thyroid storm and maternal congestive heart failure [16].

The Maltese archipelago is an independent country located in the Mediterranean Sea, 93 km south of Sicily. The islands together make up a total area of just 316 km ^2^ and have one of the densest populations in the world. According to Eurostat, the population of the Maltese Islands in 2006 amounted to 405,308 residents, increasing to 455,356 residents in 2016. The prevalence of thyroid disease in the Maltese population, in particular women of reproductive age, has not been investigated at a population level. However, data from a small observational study suggests that the prevalence stands at 17.1% in non- pregnant patients with Type 2 diabetes, an observation that is likely to be particularly relevant given that diabetes occurs in at least 10% of the Maltese population [17].

Furthermore, a pilot study involving 160 Maltese pregnant women has revealed a relatively high frequency of isolated hypothyroxinaemia (31% of the cohort) with slightly low T4 levels but normal TSH levels [18].

The level of dietary iodine intake amongst the Maltese population in particular women of reproductive age is unknown. Furthermore, in Malta there exists no national salt iodisation programmes nor any legislation requiring retailers to sell only iodised salt such as exists in Italy and which would help increase dietary iodine intake [19].

## Method

The aim of this study was to establish the prevalence of existing thyroid disorders in the Maltese pregnant population. It is a retrospective study of all births delivered in Malta between 2006 and 2016 and is based on data collected and stored by the National Obstetrics Information Service (NOIS) of the Department of Health Information and Research (DHIR). This database carries information regarding all the hospital obstetric deliveries registered in the Maltese Islands. Data is collected from all the public and private owned maternity units on both islands. Women with thyroid dysfunction identified by NOIS prior to June 2012 were those with known thyroid disease in the current or previous pregnancy, or women who were screened for thyroid dysfunction on clinical grounds during their index pregnancy. All pregnant women presenting for their routine antenatal booking since June 2012 were screened for thyroid dysfunction, in accordance with local practice recommendations.

Demographic data, past obstetric and medical history, current obstetric history including any antenatal complications as well as respective obstetric and neonatal outcomes of each pregnancy are recorded by staff at each respective maternity centre using a standard NOIS sheet. Information regarding certain medical conditions including thyroid disorders is also recorded in this database. Filled up NOIS data sheets are then processed and entered into the NOIS database. All data is kept in accordance with the Data Protection Act, 2001.

Cases recorded to have had some form of thyroid dysfunction at any stage during pregnancy had been identified by NOIS. Confirmation and correlation with lab results found in patients’ medical records and/or iSOFT® database system was then carried out for each patient. All patients included in this study had their thyroid profile analysed using the Immulite platform.

Since recommended reference ranges for TSH and T4 levels in pregnancy are not available for the local population, the laboratory TSH reference ranges recommended for the general population at the respective time of testing were adopted. Thus, these were as follows:

- Between January 2006- June 2010: -
- TSH 0.4–4.0 mIU/L
- fT4 11.5–22.7 pmol/L
- From June 2010 - December 2016
- TSH 0.3-3.0 mIU/L
- fT4 11.0–18.0 pmol/L

Review of laboratory results enabled classification of thyroid status into, normal, hypothyroidism, hyperthyroidism, isolated hypothyroxinaemia and thyroid carcinoma: -

1. Hypothyroidism- High TSH and low or normal T4.
2. Hyperthyroidism – Low TSH and high T4.
3. Isolated hypothyroxinaemia – Normal TSH and low T4.
4. Thyroid carcinoma - histology result.

Records of patients noted to have some form of thyroid dysfunction were also checked for any history of thyroid autoantibody testing and any positive or negative respective results were recorded on the study database.

Routine demographic data, past obstetric and medical history, and obstetric and neonatal outcomes in index pregnancies was analysed in pregnant patients with hypothyroidism, hyperthyroidism and isolated hypothyroxinaemia and compared to data pertaining to pregnancies in euthyroid patients. Using the Statistical Package for the Social sciences (SPSS®) version, Chi square/Fisher’s exact test were used to compare categorical variables. One-way ANOVA was used to compare continuous variables satisfying parametric assumptions while the Mann-Whitney U test was used to compare continuous variables which did not satisfy parametric assumptions. Statistical significance was defined by a two-sided *p* value < 0.05.

There was a total of 46,294 deliveries occurring between January 2006 and December 2015. 9 patients had a wrong personal identification number or missing/ incomplete data, impeding proper cross-confirmation of results. Therefore, the total number of patients in our data set was 46,283. 3,437 deliveries happened to non- Maltese nationals.

494 deliveries were originally identified by NOIS to have occurred in patients with some form of thyroid dysfunction during pregnancy. Following further confirmation/ correlation with patients’ medical records and/or lab results the total number of patients with pregnancies affected by thyroid dysfunction increased to 587. This amounts to 1.3% of all the pregnancies in the time frame studied.

## Results

The number of euthyroid patients and the number of patients suffering from the various forms of thyroid dysfunction in our cohort are summarised in Table 1.

**Table 1:** Maternal thyroid categories

Maternal thyroid category	*N* = 46,283
Euthyroid	45,696 (98.73%)
Hypothyroid	395 (0.85%)
Hyperthyroid	19 (0.04%)
Isolated Hypothyroxinaemia	166 (0.36%)
Thyroid carcinoma	7 (0.02%)

The mean ± SD age of the pregnant mothers in this data set was 29.2 ± 5.4 years. Patients with hypothyroidism and isolated hypothyroxinaemia were statistically older, as outlined in Table 2.

**Table 2:** Mean (SD) age for maternal thyroid categories

**Maternal thyroid category**	**Mean (± SD) age** **(years)**	***p* value **^**a**^** for the comparison vs euthyroid patients**
Euthyroid	29.2 (± 5.4)	
Hypothyroidism	31.0 (± 5.2)	< 0.001^a^
Hyperthyroidism	30.7 (± 5.7)	0.747^a^
Isolated hypothyroxinaemia	31.4 (± 5.1)	< 0.001^a^

^a^ ***two-tailed p value [one-way ANOVA]***

Out of all hypothyroid pregnancies (*n* = 395), 95 were positive for anti-thyroid peroxidase antibodies (TPO), 102 were negative for anti-TPO. No data was available for 198 patients.

Pregnant subjects with hypothyroidism and isolated hypothyroxinaemia in our data set were found to have significantly higher baseline Body Mass Index (BMI) than euthyroid patients, as outlined in Table 3.

**Table 3:** Body mass index (BMI) values across maternal thyroid categories

Maternal thyroid category	Mean (± SD) BMI (kg/m^2^)	*P* value for the comparison vs euthyroid patients ^a^
Euthyroid	24.8 (± 5.1)	
Hypothyroid	**26.4 (± 6.8)**	**0.001**
Hyperthyroid	24.1(± 5.6)	0.947
Isolated hypothyroxinaemia	**25.9 (± 5.1)**	**0.035**

^a^ two-tailed *p* value [one-way ANOVA]; Analysis performed on Ln_e_ transformed data

In our cohort we found no statistically significant differences in rates of past history of stillbirth or miscarriages between euthyroid and the different categories of thyroid dysfunction. Likewise rates of antepartum complications affecting index pregnancy namely threatened miscarriage, threatened pre-term labour, antepartum haemorrhage, gestational hypertensive disorders including pre-eclampsia and eclampsia, placenta praevia and placental abruption were the same for euthyroid patients and patients with thyroid dysfunction.

Interestingly patients with hypothyroidism and isolated hypothyroxinaemia were more likely to have a history of delivery by Lower Segment Caesarean Section ( LSCS) in previous pregnancies when compared to euthyroid patients. This difference reached statistical significance for both thyroid dysfunction subgroups, as outlined in Table 4.

**Table 4:** History of Lower Segment Caesarean Section, stratified by thyroid category

Maternal thyroid category	Number of patients with history of LSCS (total number of patients for whom data available)	*p* value for the comparison vs euthyroid patients
Euthyroid	6004 (45,704)	
Hypothyroidism	66 (395)	0.043^a^
Hyperthyroidism	3 (19)	0.731^b^
Isolated hypothyroxinaemia	34 (166)	0.006^a^

^a^ two-tailed *p* value [Chi Square test]

^b^ two-tailed *p* value [Fisher’s exact test]

**Fig. 1:**
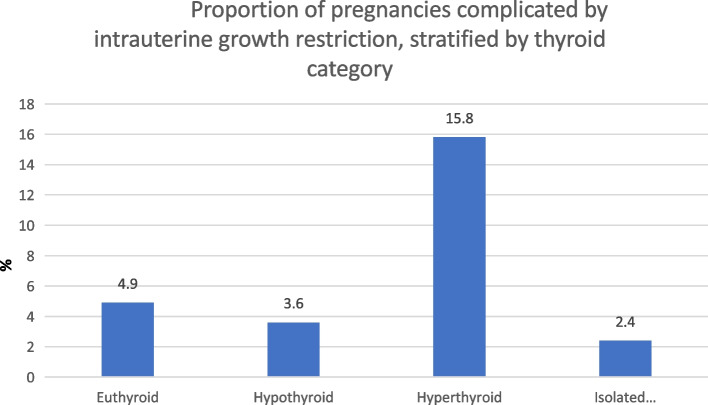
Proportion of pregnancies complicated by intrauterine growth restriction, stratified by thyroid category

Albeit not statistically significant, hyperthyroid patients in our cohort were more likely to have pregnancies complicated by intrauterine growth restriction of the foetus. As outlined in Figs. 1 15.8% of all hyperthyroidism patients had suspected intra-uterine growth restriction in comparison to 4.9% of all euthyroid patients, 3.6% of hypothyroid patients and 2.4% of patients in the isolated hypothyroxinaemia subgroup.

Patients with hypothyroidism were more likely to suffer from T1DM and GDM when compared with euthyroid patients, as outlined in Tables 5 and 6.

**Table 5:** Type 1 diabetes (T1DM) patients stratified by thyroid category

Maternal thyroid category	Number of T1DM patients (total number of patients for whom data available)	*p* value for the comparison vs euthyroid patients ^a^
Euthyroid	164 (45,586)	
Hypothyroidism	8 (394)	< 0.001
Hyperthyroidism	0 (19)	1.00
Isolated hypothyroxinaemia	1 (166)	0.452

^a^ two-tailed *p* value [Fisher’s exact test]

**Table 6:** Gestational diabetes (GDM) patients stratified by thyroid category

Maternal thyroid category	Number of gestational diabetes patients (total number of patients for whom data available)	*p* value for the comparison vs euthyroid patients
Euthyroid	1482 (45,578)	
Hypothyroidism	22 (393)	0.012^a^
Hyperthyroidism	1 (19)	0.467^b^
Isolated hypothyroxinaemia	7 (166)	0.505^a^

^a^ two-tailed *p* value (Chi Square test)

^b^ two-tailed *p* value [Fisher’s exact test]

**Fig. 2:**
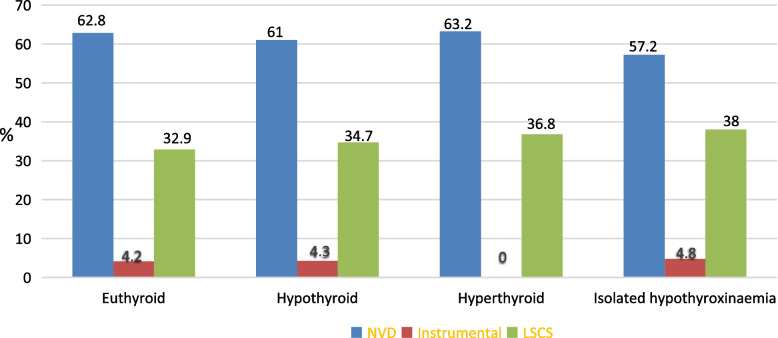
Modes of delivery, stratified by thyroid category

In our cohort we also looked at the mode of delivery of patients with normal thyroid function and patients within the various thyroid dysfunction categories. Modes of delivery were subdivided into normal vaginal delivery, instrumental delivery (forceps and vacuum assisted vaginal deliveries) and lower segment Caesarean sections). We report no significant difference in modes of delivery across thyroid categories (Fig. 2).

We report no statistically significant differences in the mean gestational age at delivery of infants born to euthyroid, hypothyroid, hyperthyroid patients and patients with isolated hypothyroxinaemia. A trend for a somewhat lower gestational age was however noted for infants born to hyperthyroid mothers, as outlined in Table 7.

**Table 7:** Gestational age across maternal thyroid categories

Maternal thyroid category	Mean (± SD) gestational age (weeks)	*P* value for the comparison vs euthyroid patients ^a^
Euthyroid	38.6 (± 1.90)	
Hypothyroid	38.7 (± 1.50)	0.445
Hyperthyroid	37.7 (± 3.2)	0.481
Isolated hypothyroxinaemia	38.7 (± 1.5)	0.718

^a^ two-tailed *p* value [Mann–Whitney]

In keeping with this trend, hyperthyroid patients were noted to have the highest percentage of patients with early pre-term delivery at less than 34 weeks (Table 8). 15.8% of hyperthyroid patients vs. 2.9% of euthyroid patients vs. 1.3% of hypothyroid patients and 1.2% of patients with isolated hypothyroxinaemia. This was statistically significant (*p* = 0.017).

**Table 8:** Preterm deliveries across maternal thyroid categories

Maternal Thyroid category	Number of patients with pre-term delivery at < 34 weeks (total number of patients for whom data available)	*P* value for the comparison vs euthyroid patients ^a, b^
Euthyroid	1328 (45,680)	-
Hypothyroid	5 (395)	0.067
Hyperthyroid	**3 (19)**	**0.017**
Isolated Hypothyroxinaemia	2 (166	0.248

^a^ two-tailed *p* value (Chi Square test)

^b^ two-tailed *p* value [Fisher’s exact test]

Mean birth weight in grams at delivery for infants of mothers in each respective thyroid dysfunction category was calculated and compared to mean birth weight of infants born to euthyroid mothers. These results are outlined in Table 9. Infants born to mothers with isolated hypothyroxinaemia in our cohort had a statistically significant higher mean birth weight (Mean birth weight +/-SD 3305.1 +/-460.9 g for isolated hypothyroxinaemia vs. 3206.2+/-535.1 g for euthyroid (p.009).

**Table 9:** Birth weight at delivery across maternal thyroid categories

Maternal thyroid category	Birth weight at delivery (g) (Mean + /_ SD)	*P* value for the comparison vs euthyroid patients
Euthyroid	3206.2 ± 535.1	
Hypothyroidism	3257.7 ± 502.7	0.140
Hyperthyroidism	3033.2 ± 854.7	0.573
Isolated hypothyroxinaemia	**3305.1 ± 460.9**	**0.009**

^a^ two-tailed *p* value [Mann–Whitney]

We report no significant differences in background rates of post-partum haemorrhage including the need for hysterectomy in the first 48 h post- partum, retained placenta and shoulder dystocia between euthyroid patients and patients in each thyroid dysfunction sub category.

## Discussion

The total number of pregnancies affected by thyroid dysfunction amounted to only 1.3% of all pregnancies within the time frame studied by our cohort. Given the retrospective nature of our study, we were unable to determine separate prevalences for overt and subclinical hypothyroidism respectively. However, the collective prevalence of hypothyroidism (overt and subclinical) in the Maltese pregnant population is 0.85% while that for hyperthyroidism is 0.04%. The prevalence of isolated hypothyroxinaemia was found to be 0.36%. These low prevalences seem surprising given previous data suggesting higher prevalence rates of thyroid disease in the Maltese population [17, 18].

We suspect that the low prevalence rates of thyroid dysfunction in our cohort can be explained by unreported or unrecorded cases given the retrospective nature of this study. During the period covered by this study routine antenatal screening for thyroid dysfunction at booking had not yet been fully adopted and most reported cases were known cases of thyroid dysfunction diagnosed before pregnancy. Furthermore, in the absence of recommended reference ranges for TSH and T4 levels in pregnancy for the local population, the laboratory TSH reference ranges recommended for the general population at the respective time of testing were adopted in interpreting thyroid function test results for those pregnancies diagnosed during pregnancy. This could have led to misinterpretation of some thyroid function results in pregnancy. Both the ATA and ES guidelines recommend establishing and using population and trimester specific TSH and T4 reference ranges [20, 21].

Consistent with other data [22–24], patients with thyroid dysfunction in our cohort were found to be older than euthyroid patients while patients with hypothyroidism and isolated hypothyroxinaemia had higher baseline BMIs than euthyroid patients. It has been suggested that isolated low thyroxine levels in obese patients is secondary to high BMI rather than high BMI being the result of altered thyroid function [23].

Our study failed to confirm previous reports of increased risk of gestational hypertension and pre-eclampsia, pregnancy loss, placental abruption, low Apgar scores or PPH associated with hypothyroidism. Once again, we suspect a possible explanation for our findings might be due to unreported/ undiagnosed and possibly untreated cases which would be listed as euthyroid with potential skewing of the data. was also lacking. Furthermore, data regarding what type of hypothyroidism and information regarding treatment with consequent efficacy was lacking. In addition, most patients with thyroid dysfunction lacked information regarding the presence or absence of thyroid auto antibodies. Our data set was collected during a period when there was less awareness about thyroid dysfunction in pregnancy.

Several studies and meta-analysis found that pregnant patients with overt and subclinical hypothyroidism were at a significantly higher risk of developing GDM when compared to patients with normal thyroid function [8, 9, 22]. Our study also showed an increased risk for GDM for hypothyroid pregnant patients but not for patients with isolated hypothyroxinaemia. The meta-analysis by Gong et al. also failed to show an association between isolated hypothyroxinaemia and GDM while in other studies patients with isolated hypothyroxinaemia were found to have an increased risk for GDM [23, 24].

In our cohort, patients with hyperthyroidism were found to have a significantly increased risk of early preterm delivery before 34 weeks of gestation. Albeit not statistically significant, pregnancies with this form of thyroid dysfunction were also more likely to have suspected intrauterine growth restriction and low mean birth weight. Prematurity, intra-uterine growth restriction and low birth weight have already been associated with thyrotoxicosis in previous studies [25]. Our study however failed to confirm any associations with other adverse maternal and perinatal outcomes possibly due to low numbers involved.

Although we found no significant differences as regards mode of delivery of index pregnancies between euthyroid patients and patients in the various thyroid dysfunction subcategories in our cohort, patients with hypothyroidism and isolated hypothyroxinaemia had a higher incidence of past history of delivery by lower segment caesarean sections in previous pregnancies. A number of studies reported an association between hypothyroidism and increased risk of caesarean delivery [22] while others [24] found an increased risk for caesarean sections in patients with isolated hypothyroxinaemia.

The mean birth weight of infants born to mothers with isolated hypothyroxinaemia was found to be significantly more than that of infants born to euthyroid patients. This finding is consistent with reports from other studies where isolated hypothyroxinaemia has been found to be associated with an increased risk of large for gestational age infants [24, 26].

To the authors’ knowledge, this study represents the first large scale attempt at identifying the prevalence of thyroid dysfunction in pregnancy in the Maltese setting. Its observational design permits the analysis and potential identification of rare adverse outcomes in a larger data set of patients which should be representative of the whole population. Indeed, NOIS captures data pertaining to all pregnancies occurring in the Maltese islands. While permitting efficient use of available data, this study allows identification of disease over a longer time period than would be reasonably permitted by a prospective study.

An attempt has been made to account for the possibility of incomplete/ inaccurate data (an inherent limitation of observational studies) by manually validating outcomes of interest using hospital record data. Nonetheless such a retrospective cohort study will be limited by the effect of unmeasured and/or unknown confounding variables and the possibility of reverse causation. Furthermore, the study size precludes multivariate analysis.

Given the findings of our study and the discrepancy in prevalence reported between a previous pilot study and this large retrospective study we plan to embark on a large-scale prospective study.

## Conclusion

Thyroid dysfunction among Maltese pregnant women may be unreported or insufficiently recognised. Available evidence suggests that thyroid dysfunction is associated with certain comorbidities and anthropometric characteristics and impacts on pregnancy outcomes.Further research is warranted in this field.

## Supplementary Information

**Additional file 1.**

## Data Availability

The datasets generated analysed during the current study are available in the National Obstetric Information System repository of the Malta Department of Information & Research. [https://deputyprimeminister.gov.mt/en/dhir/Pages/Registries/births.aspx.
