# Supplementary material for: Thyroid dysfunction in pregnancy - a retrospective observational analysis of a Maltese cohort
Source: BMC Pregnancy Childbirth. 2022 Dec 15;22:941. doi: 10.1186/s12884-022-05266-x (PMC9756451; doi:10.1186/s12884-022-05266-x)
Supplement: Supplementary file 1 — Additional file 1. [file 12884_2022_5266_MOESM1_ESM.pdf]

Directorate for Health Information and Research, Malta

NATIONAL OBSTETRIC INFORMATION SHEET (NOIS)

Hospital: ______________________________ ....Code: |__|__|

Case identification code ....|__|__|__|__|__|__|__|__|__|__|

(A separate and unique code is to be given for each infant delivered, according to the agreed format)

Mother's details

Identity number ....|__|__|__|__|__|__|__|__| |__|

First name .... ____

Surname .... ____

Maiden surname .... ____

Address (1) .... ____

Address (2) and Post code .... ____

Infant ID ______________________________ Infant C number _________________

Infant Name ______________________________ Infant DOB _________________

Locality of residence .... _________________________

Locality code (*office use*) ....|__|__|__|__|

Current Nationality ____.............. |__|__|__|

Migrant (0 = No, 1 = Yes, 9 = UN) ....|__|

Resident (since 1 year) (0 = No, 1 = Yes, 9 = UN) ....|__|

Country of Birth _______________________________________.................... |__|__|

Date of birth (dd/mm/yyyy) *(only the year is mandatory)* .... |__|__| |__|__| |__|__|__|__|

Legal Marital status

(1 = Single (never married), 2 = Married 3 = Widowed / separated / divorced /annulled 9 = UN) ....|__|

Mother's education – highest completed level

(1 =None, 2 =Special School, 3 =Primary,4 =Secondary, 5 =Post secondary, 6 =Non-tertiary, 7 =Tertiary, 9 =UN) ....|__|

Mother's occupation in pregnancy ____

(Office use: ISCO code) ....|__|__|__|__|

Father's Age (88 = Un-acknowledged Father, 99 = UN) .... |__|__|

Father's Nationality (if not Maltese) ______________________________..... |__|__|__|

Father's occupation .... ____

(*Office use: ISCO code*) ....|__|__|__|__|

Forms completed by ____ on _______________ .

Data is collected in accordance with Data Protection Requirements

DHIR_NOIS_v.2019

Past History (before the present pregnancy/confinement)

Past Medical History of note prior to this pregnancy (0 = No, 1 = Yes, 9 = UN) ....|__|

NIDDM – Pre-existing Non Insulin Dependant Diabetes Mellitus (0 = No, 1 = Yes, 9 = UN) ...|__|

IDDM – Pre-existing Insulin Dependant Diabetes Mellitus (0 = No, 1 = Yes, 9 = UN) ....|__|

Pre-existing Hypertension (0 = No, 1 = Yes, 9 = UN) ....|__|

Cardiovascular disease (0 = No, 1 = Yes, 9 = UN) ....|__|

Depression (0 = No, 1 = Yes, 9 = UN) ....|__|

Asthma (0 = No, 1 = Yes, 9 = UN) ....|__|

Epilepsy (0 = No, 1 = Yes, 9 = UN) ....|__|

DVT – Deep Vein Thrombosis (0 = No, 1 = Yes, 9 = UN) ....|__|

Thalassemia (0 = No, 1 = Yes, 9 = UN) ....|__|

Sickle cell Anemia (0 = No, 1 = Yes, 9 = UN) ....|__|

HIV (0 = No, 1 = Yes, 9 = UN) ....|__|

Hepatitis (Hep A, B, or C) (0 = No, 1 = Yes, 9 = UN) Specify ______________________ |__|

Other Infection including STD (0 = No, 1 = Yes, 9 = UN) Specify______________________ |__|

Other Pathology (specify below) (0 = No, 1 = Yes, 9 = UN) ....|__|

Rubella vaccination (0 = No, 1 = Yes, 9 = UN) ....|__|

Past transfusion (0 = No, 1 = Yes, 9 = UN) ....|__|

Pelvic fractures (0 = No, 1 = Yes, 9 = UN) ....|__|

Past surgery (0 = No, 1 = Yes, 9 = UN) Specify ______________________.|__|

Pre-existing Thyroid problems
(0 = No, 1=Yes, NOS, 2=Hypothyroid, 3= Hyperthyroid, 4= Other, 9 = UN) ....|__|

Female Genital Mutilation
(0 = No, 1 = Yes, Type I, 2 = Yes, Type II, 3= Yes, Type III, 4= Yes, Type IV, 5 = Yes, NOS, 9 = UN) .....|__|

Other Past Medical History of note - ____________________________________ ... |__|__|__|.|__|

(*Office use: ICD-10 code*) - ____________________________________... |__|__|__|.|__|

Past Gynae History of note prior to this pregnancy (0 = No, 1 = Yes, 9 = UN) ....|__|

Infertility (0 = No, 1 = Yes, 9 = UN) ....|__|

Previous assisted conception/ infertility treatment (0 = No, 1 = Yes, 9 = UN) ....|__|

Pelvic inflammatory disease PID (0 = No, 1 = Yes, 9 = UN) ....|__|

Postnatal depression (0 = No, 1 = Yes, 9 = UN) ....|__|

Past Gynae Surgery (0 = No, 1 = Yes, 9 = UN) Specify __________________________.............|__|

Other Past Gynae History of note (0 = No, 1 = Yes, 9 = UN) ....|__|

Specify______________________________________.. |__|__|__|.|__|

______________________________________.. |__|__|__|.|__|

Case identification code .... |__|__|__|__|__|__|__|__|__|__|

Past Obstetric History

Spontaneous miscarriages (b.wt. under 500g and/or below 22 wks gestation) (number) .... |__|__|

Induced abortions (number).. |__|__|

Ectopic pregnancies (number) ....|__|

Vaginal deliveries (number) .... |__|__|

Caesarean sections (number) .... |__|__|

Number of livebirths (in all) .... |__|__|

Number of livebirths who died in 1st week of life (early neonatal dth) .... |__|__|

Number of livebirths who died in 2nd, 3rd or 4th weeks of life (late neonatal dth) ...... |__|__|

Number of still births (b.wt. 500g or more, or gestation 22 wks or more) .... |__|__|

Number of preterm deliveries (before completing 37 wks of gestation) .... |__|__|

Complications in previous pregnancies (eg. IGT, GDM, PIH, DVT, molar preg.) _______. |__|__|__|.|__|

____.... |__|__|__|.|__|

Present Obstetric History

Last menstrual period (dd/mm/yyyy) .... |__|__| |__|__| |__|__|__|__|

Certainty of LMP – Last Menstrual Period (0 = No, 1 = Yes, certain, 9 = UN) ....|__|

Regular menstrual cycle (0 = No, 1 = Yes, 9 = UN) ....|__|

Expected date of delivery (EDD) by dates (dd/mm/yyyy) .... |__|__| |__|__| |__|__|__|__|

Expected date of delivery (EDD) by ultrasound (dd/mm/yyyy) .... |__|__| |__|__| |__|__|__|__|

Planned Pregnancy (0 = No, 1 = Yes, 9 = UN) ....|__|

Contraception at time of current conception (0 = No, 1 = Yes, 9 = UN) ....|__|

Assisted fertilisation (0 = No, 1 = Yes, NOS, 2 = Induction of ovulation only, 3 = Artificial insemination , 4 = Implantation of ovum /ova (IVF) / ICSI, 9 = UN) ______________________________ |__|

Cigarette smoking during pregnancy (0 = None, 1= Stopped in pregnancy, 2 = still smoking, 9 = UN) ..|__|

No. of cigs per day ....|__|

Alcohol use during this pregnancy (0 = None, 1= Stopped in pregnancy, 2 = still drinking, 9 = UN) ...|__|

No. of units per week ....|__|

Illicit Drug abuse during pregnancy (0 = No, 1 = Yes, 9 = UN) Specify ________________ ...|__|

Non prescribed medicinal drug **abuse** during pregnancy (0 = No, 1 = Yes, 9 = UN) ....|__|

Drugs or Medications Abused ______________________......................|__|__|__|__|__|__|__|

(*Office use: ATC - code*) ______________________ ....|__|__|__|__|__|__|__|

Medications during 1st trimester of pregnancy

Folic Acid. (0 = No, 1 = Yes NOS, 2 = Yes started before pregnancy, 3 = Yes started in 1st trimester, 4 = Yes started after 1st trimester, 9 = UN) ....|__|

Iron Supplements (0 = No, 1 = Yes NOS, 2 = Yes started before pregnancy, 3 = Yes started in 1st trimester, 4 = Yes started after 1st trimester, 9 = UN) ....|__|

Multivitamins (0 = No, 1 = Yes NOS, 2 = Yes started before pregnancy, 3 = Yes started in 1st trimester, 4 = Yes started after 1st trimester, 9 = UN) ....|__|

Chronic Medication History of note in 1st trimester (0 = No, 1 = Yes, 9 = UN) ....|__|

Infertility treatment – (G03G) (0 = No, 1 = Yes, 9 = UN) ....|__|

Progestogens – (G03D) (0 = No, 1 = Yes, 9 = UN) ....|__|

Aspirin – (B01AC06) (0 = No, 1 = Yes, 9 = UN) ....|__|

Antiemetics – (A04) (0 = No, 1 = Yes, 9 = UN) ....|__|

Antiasthmatics – (R03) (0 = No, 1 = Yes, 9 = UN) ....|__|

Antihypertensives – (C02) (0 = No, 1 = Yes, 9 = UN) ....|__|

Antidiabetics – (A10) (0 = No, 1 = Yes, 9 = UN) ....|__|

Antiepileptics – (N03) (0 = No, 1 = Yes, 9 = UN) ....|__|

Antidepressants – (N06A) (0 = No, 1 = Yes, 9 = UN) ....|__|

Thyroxine – (H03AA01) (0 = No, 1 = Yes, 9 = UN) ....|__|

Antibiotics – (J01) (0 = No, 1 = Yes, 9 = UN) ....|__|

Other medication in 1st Trimester (0 = No, 1 = Yes, 9 = UN) ....|__|

Specify 1 - ______________________ ....|__|__|__|__|__|__|__|__|

(*Office use: ATC - code*) 2 - ______________________ ....|__|__|__|__|__|__|__|__|

Antenatal Examination:

Date of First antenatal visit (dd/mm/yyyy) .... |__|__| |__|__| |__|__|__|__|

Gestation at First antenatal visit (week of gestation) 99 = UN .... |__|__|

Number of Antenatal visits (if exact number is uncertain <4 = 33, >=4=44, UN=99) .... |__|__|

Height (nearest cm) (999 = UN).. |__|__|__|

Weight before pregnancy (nearest kg) (999 = UN).. |__|__|__|

Weight at delivery (nearest kg) (999 = UN) .... |__|__|__|

Ultrasound scans during pregnancy

Number of scans (if exact number is uncertain <4 = 33, >=4=44, UN=99)................................ |__|__|

First recorded scan (week of pregnancy) (99 = UN) .... |__|__|

Last recorded scan (week of pregnancy) (99 = UN).. |__|__|

Detailed anomaly scan for malformations (0 = No, 1 = Yes, 9 = UN) ....|__|

Fetal Anomalies detected by US (0 = No, 1 = Yes, 9 = UN) Specify ____________________ ..|__|

If yes – Date at first anomaly detection US (dd/mm/yyyy) .... |__|__| |__|__| |__|__|__|__|

Gestation at first anomaly detection by US (in weeks) .... |__|__|

Multiple gestation detected (0=No, 1=Yes, NOS, 2=Yes, monochorionic, 3=Yes, dichorionic, 9=UN) .... |__|__|

If yes – Gestation at first diagnostic US (in weeks).. |__|__|

Screening in pregnancy:

Thalassemia (0 = No, 1 = Yes, result +ve, 2 = Yes, result –ve, 3 = Yes, result nk, 9 = UN) .... |__|

Sickle cell (0 = No, 1 = Yes, result +ve, 2 = Yes, result –ve, 3 = Yes, result nk, 9 = UN) .... |__|

Hepatitis B (0 = No, 1 = Yes, result +ve, 2 = Yes, result –ve, 3 = Yes, result nk, 9 = UN) .... |__|

Hepatitis C (0 = No, 1 = Yes, result +ve, 2 = Yes, result –ve, 3 = Yes, result nk, 9 = UN) .... |__|

HIV (0 = No, 1 = Yes, result +ve, 2 = Yes, result –ve, 3 = Yes, result nk, 9 = UN) .... |__|
If yes – Anti viral treatment given: (0 = No, 1 = Yes, 9 = UN) .... |__|

Syphilis (0 = No, 1 = Yes, result +ve, 2 = Yes, result –ve, 3 = Yes, result nk, 9 = UN) .... |__|
If yes – treatment given: (0 = No, 1 = Yes, 9 = UN) .... |__|

Grp B Strep (0 = No, 1 = Yes, result +ve, 2 = Yes, result –ve, 3 = Yes, result nk, 9 = UN) .... |__|
If yes – treatment given: (0 = No, 1 = Yes, 9 = UN) .... |__|

Other Screening Specify: ______________________________________ ...... |__|__|__|.|__|

Immunity Status in pregnancy:

Rubella IgG +ve |__|, -ve |__|; IgM +ve |__|, -ve |__|; UN |__|

Varicella IgG +ve |__|, -ve |__|; IgM +ve |__|, -ve |__|; UN |__|

Other (eg toxoplasma) Specify: ___________________________________ |__|__|__|.|__|

Confirmed Pathology during pregnancy (0 = No, 1 = Yes, 9 = UN) .... |__|

Threatened abortion (before 22 wks gestation) (0 = No, 1 = Yes, 9 = UN) .... |__|

Premature spontaneous labour (22 - 37 wks.) (0 = No, 1 = Yes, 9 = UN) .... |__|

Antepartum haemorrhage (APH) (0 = No, 1 = Yes, 9 = UN) .... |__|

PIH (persistently > 140/90mmHg, no albuminuria) (0 = No, 1 = Yes, 9 = UN) .... |__|

Pre-eclampsia (hypertension with albuminuria) (0 = No, 1 = Yes, 9 = UN) .... |__|

Eclampsia (severe hypertension with seizures) (0 = No, 1 = Yes, 9 = UN) .... |__|

Placenta praevia (0 = No, 1 = Yes, 9 = UN) .... |__|

Abruption of placenta (0 = No, 1 = Yes, 9 = UN) .... |__|

Suspected IUGR –Intrauterine Growth Retardation (0 = No, 1 = Yes, 9 = UN) .... |__|

Blood group immunisation (0 = No, 1 = Yes, 9 = UN) .... |__|

Anaemia requiring transfusion (0 = No, 1 = Yes, 9 = UN) .... |__|

Units transfused in pregnancy (9 = UN) .... |__|

Cardiovascular disease (0 = No, 1 = Yes, 9 = UN) .... |__|

Acute Hepatitis (0 = No, 1 = Hepatitis A, 2 = Hepatitis B, 3 =Hepatitis C, 4 = Other, 9 = UN) .... |__|

Urinary Tract Infection (UTI) (0 = No, 1 = Yes, 9 = UN) .... |__|

Sexually Transmitted Disease (incl. Syphilis, HPV): (0 = No, 1 = Yes, 9 = UN) .... |__|__|__|.|__|

Hypothyroidism and Diabetes appearing during this pregnancy

Hypothyroidism diagnosed during this pregnancy (0=No, 1=Yes, 9=UN) ....|__|

Gestational Diabetes / Impaired glucose tolerance (0 =No, 1 =Yes, 9 =UN) ....|__|

Other pathology in pregnancy (specify below) (0 =No, 1 =Yes, 9 =UN) ....|__|

If diabetic before or during pregnancy:

OGTT – Oral Glucose Tolerance Test done (0 = No, 1 = Yes, 9 = UN) ....|__|

If yes: OGTT level at 2 hours (mmol/l)..|__|__|.|__|

HbA1c test done (0 = No, 1 = Yes, 9 = UN) ....|__|

If yes: result in 1st trimester............................ |__|__|__| mmol/mol or |__|__| %

Insulin treatment given sometime during pregnancy (0 = No, 1 = Yes, 9 = UN) ....|__|

Other pathology complicating pregnancy:

(*Office use: ICD-10 code*) 1 - ________________________________ .....**O** |__|__|.|__|

2 - ________________________________ .....|__|__|__|.|__|

Details on Delivery

Date of delivery (dd/mm/yyyy) .... |__|__| |__|__| |__|__|__|__|

Site of delivery (1 = Hospital/clinic, 2 = Home , 3 = Underwater, 4 = Other site, 9 = UN) ....|__|

Onset (1= Induced, medical and/or surgical, 2 = Spontaneous onset, 3 = CS (Caesarean Section) elective / planned, 4 = CS emergency for pathological condition eg APH, PET, fetal distress, 9 = UN) ....|__|

Any augmentation medical and/or surgical (0 = No, 1 = Yes, 9 = UN) ....|__|

Reason for Induction_______________________________________ ....|__|__|__|.|__|

Delivery (1 = Unassisted, vaginal, 2 = Forceps, 3 = Ventouse, 4 = Breech, 5 = Elective CS before labour, 6 = Emergency CS before labour, 7 = Elective CS during labour, 8 = Emergency CS during labour, 9 = UN) .....|__|

Reason for Caesarean Section_______________________________________.....|__|__|__|.|__|

Presentation at Delivery (1 = Vertex, 2 = Breech, 3 = Transverse, 4 = Unstable, 5 = Other, 9= UN)..........|__|

Most senior attendant (0 = Unattended, 1 = Midwife/nurse, 2 = Foundation doctor, 3 = BST/HST, 4 = Resident Specialist, 5 = Consultant, 6 = General Practitioner, 7 = Other, 9 = UN) ....|__|

Midwife in charge of delivery (Reg No) (9999 = UN)...|__|__|__|__|

Consultant responsible for delivery (Med Reg No) (9999 = UN).................................|__|__|__|__|

Corticosteroids given (0 = No, 1 = Yes, 9 = UN) ....|__|

Analgesia (0 = None, 1 = Inhalation, 2 = Opioid, 3 = inhalation & opioid, 4 = Regional, 5 = inhalation & regional, 6 = opiod & regional, 7 = inhalation, opiod and regional, 8 = Other, 9 = UN) ....|__|

Anaesthesia (0 = None, 1 = General (GA), 2 = Epidural/caudal, 3 = Spinal, 4 = epidural and spinal, 5 = Epidural and general , 6 = Spinal and general, 8 = Other, 9 = UN) ....|__|

Rupture of membranes for more than 24 hours (0 = No, 1 = Yes, 9 = UN) ....|__|

Length of Stage 1 .... |__|__| hrs |__|__| mins

Length of Stage 2 .... |__|__| hrs |__|__| mins

Details on Delivery (cont.)

Perineal trauma /damage (0 = No, 1 = Yes, 9 = UN) ....|__|

Episiotomy (0 = No, 1 = Yes, 9 = UN) ....|__|

Tears (0 = No, 1 = 1st degree, 2 = 2nd degree, 3 = 3rd degree, 4 = 4th degree, 5 = Tear, not specified, 9 = UN) ......|__|

Damage to cervix (0 = No, 1 = Yes, 9 = UN) ....|__|

Hysterectomy after less than 48 hours (0 = No, 1 = Yes, 9 = UN) ....|__|

Retained placenta /membranes (0 = No, 1 = Yes, 9 = UN) ....|__|

Bleeding > 1000ml within 2 hrs (0 = No, 1 = Yes, 9 = UN) ....|__|

Blood transfusion (units) ....|__|__|

Haemoglobin level 7 g/dl or less (0 = No, 1 = Yes, 8 = Not applicable, 9 = UN) ....|__|

Shoulder dystocia (0 = No, 1 = Yes, 9 = UN) ....|__|

Maternal Eclamptic Seizures (0 = No, 1 = Yes, 9 = UN) ....|__|

Surgery (exclude tubal ligation or CS) (0 = No, 1 = Yes, 9 = UN) ....|__|

Embolisation (0 = No, 1 = Yes, 9 = UN) ....|__|

Transfer from Delivery Unit (1 = Postnatal, 2 = HDU/ITU, 3 = Gynae ward, 4 = Home, 5 = Other, 9 = UN) |__|

High Dependency Unit HDU / ITU required (0 = No, 1 = Yes, 9 = UN) ....|__|

Other special conditions during delivery ......______________________________ |__|__|__|.|__|

(*Office use: ICD-10 code*) ....________________________________|__|__|__|.|__|

Maternal discharge (1 = Home, 2 = Transferred to health facility, 3 = Dead, 4 = Other, 9 = UN) ....|__|

Discharge days or hours after delivery (99 = UN) ....|__|__| days |__|__| hours

Infant Details

Birth order number (1 of 1, except in multiple gestation) ....|__| of |__|

Date of birth (dd/mm/yyyy) .... |__|__| |__|__| |__|__|__|__|

Time of birth (hh:mm, 24 hr clock) ....|__|__|:|__|__|

Sex (1 = Male, 2 = Female, 3 = Indeterminate, 9 = UN) ....|__|

Birth weight (grams) (9999 = UN) ....|__|__|__|__|

Best estimate of Gestation at delivery (in weeks; 99 = UN) ....|__|__|

Head circumference (nearest cm; 99 = UN) ....|__|__|

Cord blood pH at delivery (88 = not taken, 99 = UN)...|__|__|

Apgar score at 1 minute (99 = UN)..|__|__|

Apgar score at 5 minutes (99 = UN) ....|__|__|

Breast feeding at delivery unit (within first hour) (0 = No, 1 = Yes, 2 = Baby died, 9 = UN) ....|__|

Infant Details (Cont.)

Infant morbidity (0 = No, 1 = Yes, 9 = UN) .... |__|

RDS/Hyaline membrane disease (0 = No, 1 = Yes, 9 = UN) .... |__|

Other respiratory conditions (0 = No, 1 = Yes, 9 = UN) .... |__|

Seizure while at hospital (0 = No, 1 = Yes, 9 = UN) .... |__|

Hyperbilirubinaemia (needing phototherapy) (0 = No, 1 = Yes, 9 = UN) .... |__|

Sepsis (confirmed - positive blood culture) (0 = No, 1 = Yes, 9 = UN) .... |__|

Other infant morbidity (specify below) (0 = No, 1 = Yes, 9 = UN) .... |__|

Transfer from Delivery unit (1 = Nursery, 2 = NIPCU, 3 = Other hospital, 4 = Died, 5 = Other, 9 = UN) .... |__|

Length of NPICU stay (days) (88= not applicable, 99 = UN) .... |__|__|

Use of surfactant (0 = No, 1 = Yes, 9 = UN) .... |__|

Mechanical Ventilation or CPAP (0 = No, 1 = Yes, 9 = UN) .... |__|

Congenital malformations (most serious)............______________________________ **Q** |__|__|.|__|

(*Office use: ICD-10 code*) ....______________________________ |__|__|__|.|__|

Other perinatal pathology (incl. infections)..........______________________________ **P** |__|__|.|__|

(*Office use: ICD-10 code*) ....______________________________ |__|__|__|.|__|

Feeding method at discharge (1 = Breast only, 2 = Bottle only, 3 = Mixed, 4 = Died, 8 = Other, 9=UN) .. |__|

Discharge days after birth (99 = UN) .... |__|__|

Method of infant discharge (1 = Home/Crèche, 2 = Transferred to health facility, 3 = Dead,
4 = Still in hospital after 28 days, 9 = UN) .... |__|

Outcome (1 = Born alive and lived at least 4 weeks, 2 = Antepartum stillbirth (no heart beat on admission),
3 = Intrapartum stillbirth (heart beat heard on admission), 4 = Stillbirth, not known whether AP or IP, 5 = Died in 1st week of life, 6 = Died in 2nd, 3rd or 4th week of life)... |__|

Date of death (if applicable - dd/mm/yyyy) .... |__|__| |__|__| |__|__|__|__|

Cause of death (1 = Congenital malformations, 2 = Pregnancy-related disorders, 3 = Birth asphyxia/trauma,
4 = Immaturity-related disorder, 5 = Infection, 6 = Other, 8 = Baby alive, 9 = UN) .... |__|

(*Office use: Underlying cause of death (UCD) ICD-10 code*) .... |__|__|__|.|__|

Hours lived before death.. |__|__|__| hours |__|__| mins

COMMENTS

____

____

____
